# APASL consensus statements and recommendations for hepatitis C prevention, epidemiology, and laboratory testing

**DOI:** 10.1007/s12072-016-9736-3

**Published:** 2016-05-26

**Authors:** Masao Omata, Tatsuo Kanda, Lai Wei, Ming-Lung Yu, Wang-Long Chuang, Alaaeldin Ibrahim, Cosmas Rinaldi Adithya Lesmana, Jose Sollano, Manoj Kumar, Ankur Jindal, Barjesh Chander Sharma, Saeed S. Hamid, A. Kadir Dokmeci, Mamun Al-Mahtab, Geofferey W. McCaughan, Jafri Wasim, Darrell H. G. Crawford, Jia-Horng Kao, Osamu Yokosuka, George K. K. Lau, Shiv Kumar Sarin

**Affiliations:** 1 Yamanashi Prefectural Central Hospital, 1-1-1 Fujimi, Kofu-shi, Yamanashi 400-8506 Japan; 2 The University of Tokyo, 7-3-1 Hongo, Bunkyo-ku, Tokyo, 113-8655 Japan; 3 Graduate School of Medicine, Chiba University, Chiba, Japan; 4 Peking University People’s Hospital, Peking University Hepatology Institute, Beijing, China; 5 Kaohsiung Municipal Ta-Tung Hospital, Kaohsiung, Taiwan; 6 Hepatobiliary Division, Department of Internal Medicine, Kaohsiung Medical University Hospital, Kaohsiung Medical University, Kaohsiung, Taiwan; 7 GI/Liver Division, Department of Internal Medicine, University of Benha, Banha, Egypt; 8 Digestive Disease and GI Oncology Center, Medistra Hospital, University of Indonesia, Jakarta, Indonesia; 9 University Santo Tomas Hospital, Manila, Philippines; 10 Department of Hepatology, Institute of Liver and Biliary Sciences, New Delhi, India; 11 Department of Gastroenterology, G.B. Pant Hospital, New Delhi, India; 12 Department of Medicine, Aga Khan University and Hospital, Stadium Road, Karachi, 74800 Pakistan; 13 Department of Gastroenterology, Ankara University School of Medicine, Ankara, Turkey; 14 Department of Hepatology, Bangabandhu Sheikh Mujib Medical University, Dhaka, 1000 Bangladesh; 15 Royal Prince Alfred Hospital, Centenary Institute, University of Sydney, Sydney, Australia; 16 University of Queensland, School of Medicine, Woolloongabba, QLD 4102 Australia; 17 National Taiwan University College of Medicine and National Taiwan University Hospital, Taipei, Taiwan; 18 The Institute of Translational Hepatology, Beijing 302 Hospital, Beijing, China

## Abstract

The Asian Pacific Association for the Study of the Liver (APASL) convened an international working party on “APASL consensus statements and recommendations for management of hepatitis C” in March 2015 to revise the “APASL consensus statements and management algorithms for hepatitis C virus infection” (Hepatol Int 6:409–435, 2012). The working party consisted of expert hepatologists from the Asian–Pacific region gathered at the Istanbul Congress Center, Istanbul, Turkey on 13 March 2015. New data were presented, discussed, and debated during the course of drafting a revision. Participants of the consensus meeting assessed the quality of the cited studies. The finalized recommendations for hepatitis C prevention, epidemiology, and laboratory testing are presented in this review.

**Electronic supplementary material:**

The online version of this article (doi:10.1007/s12072-016-9736-3) contains supplementary material, which is available to authorized users.

## Introduction

The prevalence rates of hepatitis C virus (HCV) and HCV genotype (GT) distribution around the Asia–Pacific region were well described in a recent study [1]. Estimated HCV infection rates in the general populations were 0.1–14.7 % in the Asia–Pacific region [1]. HCV GTs vary, such as HCV GT-1, GT-2, GT-3, and other GTs, in the Asia–Pacific region [1]. The absolute numbers of currently HCV-infected both globally and in the Asia–Pacific region may recently have been scaled down, since better seroprevalence studies demonstrated lower rates of active infection in China than previously believed [2, 3]. In this article, we mainly aim to introduce the natural history of HCV infection and recent advances in hepatitis C prevention, epidemiology, and laboratory testing in Asian–Pacific countries. Grading of evidence and recommendations are shown in Supplementary Table 1.

## Natural history of HCV infection

The natural history of hepatitis C is quite variable. The majority of patients who acquire HCV do not spontaneously clear the virus and develop chronic HCV infection. Chronic infection results in liver fibrosis and ultimately cirrhosis in a subset of patients, although the rate of disease progression is variable. Patients who develop cirrhosis are at further risk for complicating events (such as variceal hemorrhage, ascites, and encephalopathy) and hepatocellular carcinoma, although many patients with compensated cirrhosis remain stable for years [4, 5].

### Acute HCV infection

Around 3–4 million people are newly infected with hepatitis C annually, and this phenomenon is not limited to developing countries, as 18,000 new HCV infections occur annually in the USA alone [6]. Populations at risk for acute hepatitis C are patients who received blood transfusions, blood products or anti-D-immunoglobulin during pregnancy prior to routine screening of blood products for HCV, intravenous drug users, intranasal cocaine users, patients with tattoos or body piercings, healthcare workers, dialysis patients, and those involved in high-risk sexual activities. The introduction of routine screening of blood products and sterile injection needles has changed the principal cohorts of newly infected patients in developed countries, although these factors are still the leading cause of newly acquired HCV infection in Asian countries [7].

Acute hepatitis C infection is infrequently diagnosed because most patients are asymptomatic. Approximately 20–30 % of adults may develop clinical symptoms, which are usually mild and nonspecific. Onset of symptoms occurs 3–12 weeks after exposure. Symptoms may include malaise, weakness, anorexia, and jaundice. Jaundice appears in only 15–20 % of acutely infected individuals, and fulminant liver failure is rare except in those with concomitant chronic hepatitis B infection [8].

HCV RNA can be detected in the serum within 1–2 weeks after exposure. The level of HCV RNA rises rapidly during the first few weeks and then peaks between 10^5^ and 10^7^ IU/mL, shortly before the peak of serum alanine aminotransferase (ALT) levels and onset of symptoms [9]. Serum ALT levels start rising 2–8 weeks postexposure and often reach levels more than 10 times the normal upper limits. Anti-HCV antibodies (anti-HCV), as detected by enzyme-linked immunosorbent assay (ELISA), become positive near the onset of symptoms, approximately 1–3 months after exposure. Up to 30 % of patients will test negative for anti-HCV at the onset of their symptoms, making anti-HCV testing unreliable for diagnosis of acute infection. Almost all patients eventually develop antibodies; however, titers can be low or undetectable in immunodeficient patients. Approximately 10–15 % of subjects may have fluctuating infection, with marked variation in the levels of ALT in association with marked changes in HCV RNA levels, including periods of HCV RNA negativity followed by reappearance of HCV RNA. In most patients, fluctuations are present only during the first 24 weeks after exposure, but they may also continue beyond 24 weeks [10].

The risk of chronic infection after hepatitis C acquisition is high because the virus has a tendency toward rapid mutation, leading to extensive viral diversity among the viral population infecting a single host. This can contribute to viral persistence because the viral diversity allows HCV to escape immune recognition [11]. In most studies, 50–85 % of patients chronically remain HCV RNA positive following infection and seroconversion, depending on the population and the source of infection. These studies are heterogeneous in terms of their study populations, and the majority are clinic-based acute hepatitis C case series using descriptive methodologies. Discrepancies in these estimates have been attributed to a number of factors. First, the asymptomatic nature of early infection means that detection of acute infection is uncommon. Second, there are currently no diagnostic tests to differentiate between acute and chronic infection. Third, the majority of HCV infections occur in marginalized populations, such as injecting drug users (IDUs), who may be difficult to recruit into studies and maintain in follow-up. Finally, the statistical methods and definitions used to determine clearance estimates vary among studies. Of those who are able to spontaneously clear HCV, most do so within 12 weeks of seroconversion, although spontaneous clearance after a longer period of follow-up has also been described [12]. The HCV RNA clearance rate is higher when clearance is defined as a single negative HCV RNA result compared with two negative HCV RNA results, which should be the preferred definition of RNA clearance [13]. High spontaneous HCV clearance in the Asia–Pacific region may be due to the high prevalence of the favorable IL28B genotype [14].

#### Effects of treatment of acute hepatitis C

The transition between acute hepatitis C and its progression to chronic HCV infection is poorly defined. Reported estimates of time to clearance range from 1–2 weeks up to 1–3 years. Although treatment is indicated in acutely infected patients likely to develop chronic HCV, the acute phase is frequently unrecognized. Peginterferon with or without ribavirin for 24 weeks induces high rates of viral clearance [15]. Response rates are lower in patients treated more than 20 weeks postinfection, and it is often recommended to initiate the therapy if HCV RNA remains positive after 12 weeks of observation. This strategy also negates the risk of loss to follow-up in at-risk patients. Newer antiviral agents may alter this thinking [4, 5].

### Chronic HCV infection

There have been extensive studies focusing on the natural course of the disease progression of chronic hepatitis C, including fibrosis progression and the development of cirrhosis and hepatocellular carcinoma (HCC).

#### Natural progression of liver disease in chronic HCV infection

The natural history of chronic HCV is described variably. Published estimates of fibrosis progression and time to cirrhosis are dependent on the study design and the patient population investigated. Retrospective studies have often been based on large tertiary referral centers and may reflect ascertainment bias because individuals with advanced liver disease are more likely to be referred. The timing of the initial infection is based on the patient’s recall of first contact with blood products or intravenous drug abuse, which can often be inaccurate. In retrospective studies, cirrhosis rates have been reported to be between 17 and 55 %, HCC rates 1 and 23 %, and liver-related death 1 and 23 % over an estimated infection period of 20–30 years [16, 17]. Additionally, estimates from retrospective studies (17–55 %) have been higher than those from prospective studies (7–16 %), possibly reflecting referral bias in the former. Even prospective studies are limited by relatively short follow-up periods and are only available in well-defined patient cohorts, thus failing to provide information on longer-term outcomes. These studies typically show lower complication rates, as shown in a cohort of female infected with hepatitis C following administration of anti-D-immunoglobulin during pregnancy. Over a follow-up period of 18–20 years, the incidence of histologically confirmed cirrhosis was reported to be between 1 and 2 %, and bridging fibrosis between 2 and 10 % [18]. An alternative approach is retrospective prospective studies in which the precise time at which past infection with acute hepatitis C occurred can be defined retrospectively and the subjects are subsequently followed prospectively. In early retrospective prospective studies, the interval from exposure ranged from 9 to 50 years, and development of cirrhosis occurred in 0.3–15 % of patients, HCC developed in 0–1.9 %, and liver-related death occurred in 0–2.8 % [19]. Cumulatively, these studies suggest that there is a variable rate of disease progression to cirrhosis and its complications, likely due to multiple factors. Genomewide association studies revealed variants associated with the progression of liver fibrosis in HCV-infected patients [20, 21]. Further studies will be needed to clarify this point.

The progression of chronic hepatitis C is accelerated in human immunodeficiency virus (HIV)-infected patients. Hepatitis C and HIV can share routes of transmission. Coinfection with HIV has a negative impact on the natural history of HCV. Among those with persistent HCV infection, HIV coinfection has been associated with higher HCV plasma RNA viral loads and, according to most studies, with more rapid progression to cirrhosis, liver failure, and HCC. In a meta-analysis of eight cohorts of HIV/HCV-coinfected individuals, Graham et al. [22] demonstrated that HIV coinfection increased the risk of cirrhosis by a factor of 2.1 and clinically decompensated liver disease by a factor of 6.1.

#### Development of cirrhosis and its complications

HCV infection causes slowly progressive disease characterized by persistent hepatic inflammation, leading to development of cirrhosis in approximately 10–20 % of patients over 20–30 years of HCV infection. However, the published data vary considerably, with reported progression rates to cirrhosis from as low as 2–3 % to as high as 51 % over 22 years [23, 24]. The progression to cirrhosis is often clinically silent, and some patients are not known to have HCV infection until they present with the complications of end-stage liver disease. Cirrhosis rates begin to become significant after 20 years of infection. The time from HCV infection to cirrhosis depends on multiple factors and cannot be predicted in an individual patient. Multiple studies have attempted to measure the time interval from infection to cirrhosis and HCC. Frequently, the initial time of infection is not known and therefore must be estimated. Individuals who contracted HCV through a single blood transfusion or surgery are able to provide more precise time intervals from infection to cirrhosis and HCC. Although the mean time to cirrhosis in chronic HCV patients is estimated at 20 years, only 10–20 % of patients will actually develop cirrhosis within this time period [25]. A systematic review of 111 studies analyzing the natural history of HCV estimated that the prevalence of cirrhosis 20 years after infection was 16 % [95 % confidence interval (CI) 14–90 %] [26]. Overall, once cirrhosis has developed, there is a 1–5 % annual risk of HCC and a 3–6 % annual risk of hepatic decompensation. Five- and ten-year survival rates of compensated cirrhosis are 90–95 and 70–80 %, respectively. The cumulative probability of an episode of clinical decompensation is 5–10 % at 1 year, increasing to 30–40 % at 10 years from the diagnosis of cirrhosis. Once decompensated cirrhosis occurs, the 5-year survival rate falls to 40–50 % [27]. Achievement of viral eradication is associated with a marked decrease in decompensation and the incidence of bacterial infection [28].

#### Development of HCC

The risk of HCC occurrence varies among HCV patients. It is a function of the degree of liver fibrosis and the time after acquisition of the infection. In a meta-analysis of 21 case–control studies, the risk for HCC increased 17-fold in HCV-infected patients compared with HCV-negative controls [29]. Virtually all cases of HCV-related HCC occur among patients with cirrhosis. Once cirrhosis is established, HCC develops at an annual rate of 1–4 % and is increased in patients with raised alpha-fetoprotein levels at baseline. Higher estimates, in the range of 5–7 %, have been reported from Japan. The higher proportion of elderly patients infected with HCV in Japan may explain the higher incidence rate in that country because older age accelerates the development of HCC. Patients with lower degrees of fibrosis exhibit HCC development at rates of 0.5–2.6 % [30]. In the Hepatitis C Antiviral Long-term Treatment against Cirrhosis (HALT-C) trial, the overall rate of HCC development in cirrhotic patients was 7 % over 4.8 years. Importantly, a significant number of patients with Ishak grade 3 or 4 fibrosis also developed HCC, raising questions regarding what defines the need for screening in cirrhotic and noncirrhotic patient cohorts.

The risk of HCC appears to be greater with HCV GT-1b compared with HCV GT-2a/2c [31]. In contrast to hepatitis B virus (HBV) infection, HCC in patients with hepatitis C occurs almost exclusively in those with cirrhosis, suggesting that cirrhosis is the major risk factor. There is also suggestive experimental evidence that HCV infection itself can promote development of HCC. In addition, obesity is an independent risk factor for HCC development in chronic hepatitis C patients [32]. HCV GT-3 is also associated with a significantly increased risk of developing cirrhosis and HCC compared with HCV GT-1 [33–36]. Recent study [37] demonstrated that patients with diabetes or HCV GT-3 infection were significantly more likely to develop HCC after sustained virologic response (SVR) in veterans with HCV infection in the USA.

#### Survival

Survival is decreased in patients with HCV, especially in those who have developed cirrhosis. Compared with uninfected patients, patients with chronic HCV infection are more likely to die at a younger age and, as expected, from liver-related causes [38, 39]. In the large prospective HALT-C trial, which included 1050 patients with advanced fibrosis or cirrhosis who were followed for a median of 5.7 years, 122 patients (12 %) died and an additional 74 patients (7 %) underwent liver transplantation [40]. In a series of 384 patients with compensated cirrhosis, the 3-, 5-, and 10-year survival rates were 96, 91, and 79 %, respectively [41]. Once decompensated cirrhosis occurred, the 5-year survival declined substantially to approximately 50 %. Survival may also be worse in patients who develop cryoglobulinemia. Causes of death among patients with HCV vary with the age group being examined. In a population study from Denmark, the primary cause of death among patients aged 20–39 years was unnatural (reported as death due to mental and behavioral disorders related to psychoactive substance use and death resulting from external causes). The 10-year risk of unnatural death in patients between the ages of 20 and 29 years was 13 %. In patients between the ages of 40 and 59 years, deaths were equally distributed among liver-related, non-liver-related, and unnatural causes. In patients 70 years of age or older, the most common causes of death were non-liver related. Patients with HCV were at increased risk of death compared with non-HCV-infected individuals at all ages, ranging from an 18-fold increase for 20–29-year-olds to a 1.6-fold increase for patients aged 70 years or greater [42]. Mortality in patients with HCV is not always related to liver disease. A population-based study from Australia found that most deaths in young patients with HCV were due to continued drug use rather than the infection [43].

#### HCV infection and extrahepatic manifestation

Some patients with chronic HCV infection may suffer from extrahepatic illnesses, with a symptomatic spectrum varying from fatigue to permanent organ damage. These illnesses resolve following SVR by antiviral treatment [4]. Interferon-free treatment appears safe and effective in HCV patients with certain extrahepatic manifestations [44, 45].

#### Hepatitis C virus genotype 3 infection and hepatic steatosis

Patients infected with HCV often have hepatic steatosis. Several studies reported that HCV GT-3 infection was associated with hepatic steatosis compared with HCV GT-1 infection [46–48]. HCV GT-3 is the most prevalent HCV GT in patients with chronic HCV infection in North and Central India, where HCV GT-3 is also associated with significant hepatic steatosis and fibrosis [49]. Among patients infected with HCV GT-3, SVR significantly reduced steatosis, but there was no change in steatosis among those without SVR [50]. Expression of steatosis-associated HCV GT-3 core protein produces intracellular lipid accumulation in primary and cultured hepatocytes [51], and polymorphisms in HCV GT-3 core protein could produce increased intracellular lipid levels [52]. These facts may indicate the direct cytopathic effect of HCV core protein.

#### Chronic HCV infection and insulin resistance/diabetes mellitus

Several studies have shown a higher prevalence of HCV antibodies in patients with diabetes mellitus than in the general population [53, 54]. HCV infection is associated with insulin resistance/diabetes mellitus in adult patients with cirrhosis undergoing assessment for liver transplantation [55]. Allison et al. [55] reported that the prevalence of diabetes mellitus was 50 % in cirrhotic patients infected with HCV, and it was 9 % in those without HCV. Although there are several basic associations between HCV infection and insulin resistance/diabetes mellitus [56, 57], it might be difficult to cure insulin resistance/diabetes mellitus in patients with HCV and advanced liver fibrosis with interferon-free regimens.

#### Chronic hepatitis C virus infection in patients with normal versus high serum aminotransferase levels

HCV infection with normal ALT is defined by detectable HCV RNA and a serum ALT concentration that is persistently within the normal range. Using this definition, approximately 25–40 % of patients with chronic HCV infection have persistently normal serum ALT. It has been proposed that the upper limit of normal may be set too high because of unrecognized fatty liver disease among apparently healthy individuals who were included in the cohorts used to establish the normal range. This is supported by a study in which a decrease in ALT levels was noted in patients with “normal” ALT levels who achieved a SVR after undergoing treatment [58].

Many anti-HCV-positive patients with normal serum ALT concentrations have an abnormal liver biopsy, although the changes are usually mild. Patients with a persistently normal ALT are more likely to be female and generally have milder disease, a lower serum HCV RNA level, and a relatively favorable prognosis, although up to 10 % of patients have bridging fibrosis, suggesting that the relatively favorable prognosis is not universal [59]. A significant proportion of patients (20–30 %) experience ALT flares that may be associated with enhanced disease progression [60].

#### Predictive models for disease progression

Many multivariate models that can help predict disease progression in individual patients have been developed. Baseline data collected as part of the HALT-C trial were used to develop predictive models for both clinical and histological outcomes. The 1050 patients in the HALT-C trial were previous nonresponders to standard interferon therapies who had advanced fibrosis on liver biopsy. Clinical outcomes were defined as an increase in Child–Pugh score to seven or greater, variceal bleeding, ascites, spontaneous bacterial peritonitis, hepatic encephalopathy, and liver-related death. The histological outcome for the study was an increase in Ishak fibrosis score of two or more points (biopsies taken at 1.5 and/or 3.5 years after randomization).

Factors predictive of clinical outcomes on multivariable analysis were elevated aspartate aminotransferase (AST)/ALT ratio, elevated total bilirubin, low albumin, low platelet count, and increasing Ishak fibrosis score. Factors predictive of histological progression were increasing body mass index, low platelet count, and hepatic steatosis [61]. In another study, clinical and laboratory variables among 247 patients with varying degrees of HCV histologic severity were analyzed. Death from liver failure, development of HCC, and liver transplantation were considered together in the statistical analysis. History of hepatic decompensation (defined as at least one episode of ascites, jaundice, hepatic encephalopathy, or gastrointestinal bleeding of variceal origin) and serum albumin concentration were independent predictors of the above outcomes. Patients without history of decompensation and serum albumin concentration greater than 4.1 mg/dL (41 g/L) had only a 3 % chance of developing one of the endpoints within 5 years versus approximately 6 % in patients with one of these factors and 40 % in patients with both factors [62].

In a third study of 455 patients who were followed for a median of 4.7 years, the only independent predictors of progression on multivariable analysis were sporadic transmission, advanced fibrosis, and low albumin [63].

#### Spontaneous resolution of chronic hepatitis C

Spontaneous resolution of chronic hepatitis C is relatively rare and has been reported in only a few longitudinal or retrospective studies. Rates of 0.5 % resolution per year per person have been observed [64]. However, HCV RNA can persist at very low levels in the serum, peripheral lymphoid cells, and brain for many years after apparent resolution of chronic hepatitis C.

#### Effect of antiviral therapy and natural history of HCV infection

Recent data demonstrate that antiviral therapy, particularly among those achieving a SVR, is associated with long-term persistence of a SVR, improved fibrosis and inflammation scores, reduced incidence of HCC, and prolonged life expectancy [4]. This reduction in the rate of progression has also been demonstrated in patients with chronic hepatitis C and cirrhosis in some studies. The impact on slowing progression is greatest in patients with a SVR, less in relapsers, and equivocal in nonresponders [4]. With the most recent approval of direct-acting antivirals (DAAs), the rates of SVR and virological cure have greatly increased across all HCV GTs for both treatment-naïve and treatment-experienced patients [4].

#### Antiviral therapy and effect on hepatic fibrosis and inflammation

Most of the studies on the effect of treatment of fibrosis have been limited by a lack of long-term follow-up after treatment versus untreated patients. Ongoing cofactors such as alcohol and metabolic syndrome are likely to play a significant role. The majority of patients (57–94 %) demonstrate marked improvements in their necroinflammation and fibrosis scores following a SVR [65–68]. However, a small minority (1–14 %) of patients demonstrated fibrosis progression following a SVR [69]. Young age and platelet count at the SVR are predictive of fibrosis regression [70]. The HALT-C study showed that long-term therapy with peginterferon did not reduce the rate of disease progression in patients with chronic hepatitis C and advanced fibrosis, with or without cirrhosis, who did not have a response to initial treatment with peginterferon and ribavirin.

Patients with HCV cirrhosis who achieve a SVR have a reduction in their portal pressure measurements compared with nonresponders [71]. Although achieving a SVR prevents development of esophageal varices in the majority of patients with compensated HCV cirrhosis, a recent prospective study showed that, although the risk of developing varices post-SVR was vastly reduced, it was not eliminated, with a small number of patients developing small esophageal varices (2/57) [72].

A meta-analysis of data from 1013 patients from three large randomized trials demonstrated that peginterferon treatment reduced inflammation and fibrosis in patients with a SVR or who relapsed, but not in nonresponders. This improvement was more prominent with the use of peginterferon than with interferon [73]. Even in patients with advanced fibrosis or cirrhosis, inflammation and fibrosis are improved with interferon monotherapy, peginterferon monotherapy, or peginterferon plus ribavirin combination therapy [74]. Overall, studies of antiviral therapy with interferon monotherapy, peginterferon monotherapy, or peginterferon plus ribavirin combination therapy demonstrated improvement in inflammation and fibrosis in patients with a SVR, to a lesser extent in relapsers, and uncertain benefit in nonresponders.

Overall, progression of liver fibrosis to cirrhosis is rare but can occur despite a SVR. Based on a large pooled dataset from 3010 naïve patients, one study estimated the prevalence at 7 % [75]. However, another study of 121 patients with pre- and posttreatment liver biopsies reported fibrosis progression in 12 % of patients after ruling out de novo infections or existence of occult HCV via polymerase chain reaction (PCR) of liver tissue [76]. The presence of significant liver comorbidities or risk factors such as alcohol consumption or fatty liver disease are likely causes for many of the cases in which progression of liver diseases occurs post-SVR. In general, the combination of more than one liver disease, for example, hemochromatosis with alcohol consumption, or chronic viral hepatitis with alcoholic liver injury, can drive fibrosis progression in HCV patients [77].

#### Antiviral therapy and effect on incidence of hepatocellular carcinoma

Among those with chronic HCV infection without advanced fibrosis or cirrhosis, the incidence of HCC is decreased in patients with a SVR and probably also in relapsers when compared with nonresponders [78]. Several studies and a meta-analysis have concluded that eradication of HCV with antiviral therapy reduces the risk of HCC in patients with chronic hepatitis C, independent of fibrosis stage [79, 80]. However, a reduction in the risk of HCC does not necessarily indicate improvement in overall survival, and interferon is less effective in patients with cirrhosis. In addition, cirrhotic patients tend to be older, and liver-unrelated mortality may be significant and obscure any potential benefit of interferon therapy. A few studies have shown that, in patients with advanced fibrosis and/or cirrhosis who achieved a SVR, the age-adjusted hazard ratio for developing HCC and death is significantly reduced. The failure to achieve a SVR was associated with a higher risk of liver-related complications, HCC, and liver-related mortality compared with those who achieved a SVR [81]. However, several studies have demonstrated no beneficial effect of interferon therapy on the prognosis of cirrhotic patients. A recent meta-analysis that included 30 studies comprising 31,528 patients from 17 countries reported a 4.6 % absolute reduction in developing HCC following a SVR [82]. Furthermore, in patients with advanced liver disease, achieving a SVR reduced the overall risk of developing HCC from 17.8 to 4.2 %, with a reduction in incidence from 3.3 % per person-year to 1.05 % (CI 0.7–1.5 %) per person-year. Prospective data extracted from the HALT-C cohort also suggested that, following a SVR, the incidence of HCC decreased from 8.8 to 1.1 % over approximately 7 years of follow-up. A recently published meta-analysis demonstrated that antiviral treatment was associated with a reduced risk of HCC in patients who attained a SVR, compared with nonresponders; the best outcomes were observed in patients treated with ribavirin-based regimes [83]. The attainment of a SVR also demonstrated prevention of the development of esophageal varices [72, 84]. There have been case reports and long-term follow-up studies that have shown development of HCC in patients with advanced hepatic fibrosis after achievement of a SVR. These observations underscore the continued risk of HCC and the need for ongoing surveillance with imaging and alpha-fetoprotein (AFP) testing in patients with chronic hepatitis C and advanced hepatic fibrosis or cirrhosis, even after a SVR. A recent study tried to risk-stratify patients further by creating a scoring system based on age, platelets, AFP, and fibrosis score [85]. Patients were identified as low, medium, or high risk depending on their score. In the low-risk group, 9 out of 657 patients developed HCC over 9 years, equating to a 0.17 % risk per annum, an incidence rate that is well below that where screening is deemed cost effective. If this score is validated, it may help identify which patients should be recommended for long-term HCC surveillance. Achievement of eradication of HCV was associated with a marked decrease of HCC [28].

#### Antiviral therapy and effect on life expectancy

It is expected that long-term durability of a SVR, improvement in fibrosis and inflammation, and a reduced incidence of HCC translate into prolonged life expectancy. Interferon treatment decreases the risk ratio for overall death and liver-related death, particularly in patients with a SVR, while the risk of liver-unrelated death remains unchanged [86, 87].

### #1 Consensus statements and recommendations on natural history of HCV infection

1. HCV can lead to persistent infection in a high proportion of infected individuals. (A1)
2. Chronic HCV infection is generally a slowly progressing disease characterized by persistent hepatic inflammation, leading to the development of cirrhosis in approximately 10–20 % of patients over 20–30 years of HCV infection. However, the published data indicate varying progression rates to cirrhosis. (A1)
3. Once cirrhosis has developed, there is a 1–5 % annual risk of HCC and a 3–6 % annual risk of hepatic decompensation. (A1)
4. Following an episode of decompensation, the risk of death in the following year is between 15 and 20 %. (A1)
5. Achievement of a SVR leads to improved mortality and reduced adverse outcomes, although fibrosis progression and de novo HCC still occur post-SVR in a small subset of patients. (A1)
6. With the availability of DAAs, the rates of SVR and virological cure have vastly increased across all HCV genotypes for treatment-naïve patients as well as for treatment-experienced patients, especially for patients with cirrhosis, where cure has been difficult and where side effects to interferon-based regimens were difficult to tolerate and often precipitated decompensation. (A1)
7. DAAs could have an impact on the complications of cirrhosis, but this feature needs to be further investigated. (C2)

## Grading of evidence and recommendations are shown in Supplementary Table 1.

## Prevention of HCV infection in Asia

The World Health Organization estimates that more than 185 million people worldwide might be infected with HCV [88]. These are crude estimates with significant differences in prevalence, from 2.0 % in South East Asia to 3.7–3.8 % in East and Central Asia [89], and the highest of approximately 15 % in Egypt [90]. The prevalence of hepatitis C infection varies significantly in different areas and different populations among or within countries, implying different routes of transmission and different strategies needed to prevent HCV transmission [91, 92].

Effective prevention of HCV can occur through the use of a prophylactic vaccine, but vaccine trials are still in early phases or progressing slowly [93]. Therefore, the current approach for the prevention of HCV infection is to reduce transmission by reducing the risk of exposure to the virus, potentially by treatment with newly developed DAAs. In addition to a universal prevention strategy to reduce transmission by blood transfusion and other unsafe medical procedures including unsafe injection, unsafe dental management, hemodialysis, acupuncture, catheters, and other medical equipment, specific risk populations should be identified to develop population-specific prevention strategies, such as for people who inject drugs (PWID) using contaminated injection equipment, people with skin or mucous membrane inoculation, or with exposure of broken skin to contaminated blood such as through use of intranasal drugs or cosmetic procedures [94]. Sexual transmission occurs particularly in the HIV coinfected population, but is at low or no risk in heterosexual couples. Meanwhile, male who have sex with male (MSM) were recently shown to be an at-risk population. Higher prevalence of HCV among HIV-positive than HIV-negative male was observed in the Asia–Pacific region [95]. The majority of acute hepatitis C in HIV-infected patients were MSM [96, 97]. Transmission from an infected mother to infant depends on the activity status of HIV coinfection during pregnancy.

Due to the few clinical manifestations of chronic HCV infection, screening is important based on the geographical region and risk population. It would be more helpful to identify HCV infection earlier than to reduce transmission and the infection pool by initiating treatment.

### Blood safety

In 1992, the introduction of second-generation anti-HCV ELISA largely eliminated HCV transmission via donated blood by screening out infected donors. Third-generation anti-HCV enzyme immunoassay (EIA) further reduced the window period by at least 1 week. Nucleic acid testing (NAT) can further reduce the risk of HCV transmission to 0.1–2.33 per million donations by mini-pool or individual donation testing [4, 98, 99].

NAT was applied to donor HCV screening in Germany in 1997. In the Asia–Pacific region, NAT has been used for blood screening in Japan since 1999, Australia and Singapore since 2000, New Zealand since 2001, Hong Kong since 2002, South Korea since 2005, Thailand since 2006, Malaysia since 2007, Taiwan since 2008, and Mainland China since 2014 [100].

HCV antigen assays, another method of detection of active HCV infection, have been commercially available for a couple of years and can detect both free antigen and antibody-combined antigen. A few studies have shown that they can shorten the window period. Therefore, this was applied in blood testing to enhance the overall effectiveness of serological HCV screening in some countries [101–103]. However, the Global Database on Blood Safety shows that testing with anti-HCV ELISA is insufficient, particularly in countries with a low human development index—only 51.3 % of units of blood are tested for HCV [104]. Blood donations are still not routinely tested for infectious markers including HCV in 39 countries. Overall, 47 % of donations are tested in laboratories without quality assurance; most were in Asia [105]. Even the testing technologies varied by country, from all technologies in Egypt to a few blood centers in India, with the number of voluntary unpaid blood donations increasing from 3.6 million in 2007 to 4.6 million in 2008.

### #2 Consensus statements and recommendations on blood safety

1. All countries must establish a national system for blood donor selection for the donation of blood or blood components. (A1)
2. Regular audit procedures should be implemented to ensure compliance at blood-testing facilities. (B2)
3. Anti-HCV antibody tests must be quality-assured with third-generation EIA or chemiluminescence immunoassay (CIA). (A1)
4. Application of HCV antigen assays and/or nucleic acid testing for the universal screening of blood products should be country specific based on availability and cost. (B2)

### Prevention in healthcare settings

HCV infections are more likely to occur in healthcare settings with low compliance with universal precautions, associated with social imbalance, inequity of income, and inequity of health, particularly in low- and middle-income countries. The risks of HCV infection in healthcare settings include unsafe injection, dental management, medical procedures, and hemodialysis, and sometimes acupuncture in certain countries or areas.

In a meta-analysis, unsafe injection practices were found to be widespread in the developing world, accounting for more than 50 % of infections. It was estimated that each person in the developing world receives 1.5 injections per year on an average. However, at least 50 % of injections were unsafe in 14 of 19 countries [106]. Moreover, 82 % of administered injections in Asia were considered unnecessary [107]. Reuse of needles and reuse of syringes remain a common practice in Bangladesh and Pakistan [108, 109]. Unsafe injections have been estimated to transmit 2.3–4.7 million HCV infections every year [110].

It was estimated that 40 % of new HCV infections are associated with unsafe injections globally [111]. The average incidence of anti-HCV seroconversion after accidental percutaneous exposure from HCV-positive source is 1.8 % [112]. Unfortunately, unsafe injections remain a problem in developing areas, where 75 % of unsafe injections are still conducted with insufficiently sterilized equipment [111]. Other risks of HCV infection involve acupuncture and needle-stick injury, which account for up to 7 % of HCV infections [4, 113].

Medical procedures associated with HCV infection are inevitable due to low compliance with universal precautions [104] including surgical procedures [114], gastrointestinal endoscopy [115], radiopharmaceuticals [116], and oncology procedures. Low compliance with universal precautions also contributed to hemodialysis-associated HCV infection [117]. Mishandling of parenteral medication vials is a major risk for HCV infection outbreaks in dialysis units. In Egypt, the HCV infection rate was as high as 50–90 % among dialysis patients [118]. The Centers for Disease Control and Prevention (CDC) recommends that all single-use injectable medications and solutions be dedicated for use on a single patient. Medications packaged as multidose should be assigned to a single patient whenever possible [119].

The occurrence of HCV infection in healthcare settings varies depending upon the frequency of injections, medical procedures, etc. and the level of compliance with universal precautions. High frequency of injections and low compliance with universal precautions is strongly associated with a high prevalence of HCV in the general population. Compliance with universal precautions varies in the Asia–Pacific region; it is lowest in Turkey at 33.6 % [120], 40 % in India [121], and 64.7 % in China [122]. The knowledge of universal precautions was inadequate in some countries in Asia [121–124]. The factors influencing compliance were irregular supply of materials, lack of other high-level disinfection equipment, and lack of training [121, 123–125].

Body piercing, tattooing, and cosmetology are also associated with potential HCV infection [126, 127]. Tattoo-associated HCV infection is one of the greatest risks in some Asian countries such as Bangladesh, India, and Japan [1]. These data show that non-healthcare transmission routes should also be noted.

### #3 Consensus statements and recommendations on prevention in healthcare settings

1. In healthcare settings, universal precautions should be followed for healthcare and cosmetology workers and strictly carried out. (A1)
2. Regular audit should be implemented for hand hygiene, safe handling and disposal of sharp objects and waste, and safe cleaning of equipment. (B2)
3. Unnecessary injections should be avoided. (B2)

### Prevention for persons who inject drugs (PWID)

PWID are important sources of HCV infection, particularly in middle- and high-income countries. Most HCV prevalence studies are based on anti-HCV because PCR-based tests are rarely used in epidemiological studies. The estimated HCV prevalence rates varied among PWID in the Asia–Pacific region. Recently, a systematic review showed that midpoint reports ranged from 36.0 % in Afghanistan to 89.8 % in Thailand. China has the largest estimated population of PWID and had midpoint estimates of anti-HCV prevalence amongst PWID of 67.0 % [128]. Due to the common transmission route of sharing contaminated injecting equipment, the prevention strategy for HCV infection should be the same as that for prevention of HIV infection [88].

“Needle and syringe” programs, including other drug-using paraphernalia, should be recommended, and “condom” programs for people who inject drugs and their sexual partners are advisable. In addition to HIV infection prevention, HCV transmission-specific strategies should be advocated. Those strategies include implementing sterile needle and syringe programs that provide low-dead-space syringes for distribution to people who inject drugs, offering peer interventions to people who inject drugs to reduce the incidence of viral hepatitis, offering opioid substitution therapy to treat opioid dependence, reducing HCV risk behavior and transmission through injecting drug use, and increasing adherence to HCV treatment [88], although a meta-analysis showed that there was insufficient evidence that interventions with needle and syringe programs were effective [129].

Another systematic review showed no clear association of HCV prevalence with duration of injection use or age of users [130]. Unfortunately, many countries do not provide sterile needle and syringe programs, and the same countries do not provide sufficient coverage even if they do have those programs. It is estimated that only 22 syringes are provided per year per person who injects drugs. In the Asia–Pacific region, Australia had by far the greatest rate of needle–syringe distribution at 202 needle–syringes per PWID per year; however, the rate of needle–syringe distribution is only 0.5 needle–syringes per PWID per year in the Middle East [131].

In addition to injected drugs, a systematic review suggested that noninjection drug use is also associated with a higher risk of HCV infection, such as sharing of inhalation equipment for cocaine [132].

### #4 Consensus statements and recommendations on HCV prevention for persons who inject drugs (PWID)

1. Because transmission of HCV via injecting drug users (IDUs) is an increasing trend in the Asia–Pacific region, effective strategies to reduce HCV transmission in this group should be explored. (B2)
2. Harm reduction including “sterile needle and syringe programs” should have increased coverage. (B2)

### Prevention of infection via sexual transmission

Based on a cross-sectional study of sexual transmission of HCV among monogamous heterosexual couples, the maximum incidence rate of HCV transmission by sex was as low as 0.07 % per year or approximately one per 190,000 sexual contacts. No specific sexual practices were related to HCV positivity among couples [133]. However, another Asian study showed that the seroprevalence rates of anti-HCV were 5.5 % in HIV-positive MSM and 0.4 % in HIV-negative MSM [134]. This result is similar to that of a community survey conducted in London, UK that showed that the seroprevalence of anti-HCV was more common in the HIV-infected population (7.7 %) than in those without HIV infection (1.2 %) [135]. Therefore, the risk of sexual transmission is strongly associated with preexisting HIV infection and unsafe sex [136], and there is low or no risk of sexual transmission of HCV among HIV-uninfected heterosexual couples.

### #5 Consensus statements and recommendations on prevention of sexual transmission

1. There is low or no risk of sexual transmission of HCV among HIV-uninfected heterosexual couples. (A2)
2. It is recommended that correct and consistent condom use be applied in MSM with HIV infection. (B2)

### Mother-to-infant transmission

Mother-to-infant transmission (also called vertical transmission) is significantly affected by coinfection with HIV. The transmission rate varied from 4 to 8 % among mothers without HIV coinfection; however, it increased to 17–25 % among mothers coinfected with HIV [137–140]. Breastfeeding was not significantly associated with transmission [141], and cesarean section did not decrease perinatal HCV transmission from mothers to infants [140]. Therefore, no specific prevention method can be recommended for this population. The safety and potential efficacy of reducing mother-to-infant transmission by recently developed DAAs or those under development, particularly in HIV coinfection, require more research.

### #6 Consensus statements and recommendations on mother-to-infant transmission

1. No specific precaution is needed for breastfeeding to prevent vertical transmission. (B2)

### Reinfection in PWID

HCV reinfection occurs commonly in PWID. PWID with previous infection with HCV clearance are half as likely to be reinfected compared with those who had not been infected previously. However, other studies suggested that previous spontaneous clearance of HCV infection might not reduce the risk of new infection [142]. Ongoing high-risk behaviors among PWID can lead to repeated exposure to HCV, resulting in reinfection [143]. Although reinfection is common, it does not always lead to persistent infection. Spontaneous clearance of HCV reinfection has also been frequently reported with a different HCV GT from that of the initial infection [142]. Reinfection rates for PWID receiving opioid substitution therapy (OST) are the most well studied. The rate of reinfection in this population is low [144].

In essence, HCV reinfection after spontaneous or treatment-induced clearance can occur. However, the rate of HCV reinfection might be as low as 0–5 cases per 100 person-years, even among persons who continue injected drug use during and after treatment [143, 145, 146]. Due to the low reinfection rate and potential clearance after reinfection, antiviral treatment should not be withheld for these populations.

### #7 Consensus statements and recommendations on prevention of reinfection in PWID

In high-risk HCV-reinfection populations (PWID), frequent testing is recommended. Education and counseling about the risk of reinfection are advised for this population. (B1)

### Reinfection following liver transplantation

Reinfection following liver transplantation has been observed, and it leads to liver disease in approximately 30 % of patients. However, newly developed DAAs make it easy to achieve a SVR both among compensated liver transplant recipients with no fibrosis or mild fibrosis [147, 148] and before liver transplantation [149].

### #8 Consensus statements and recommendations on prevention of reinfection in liver transplant recipients

Liver transplant recipients with HCV reinfection should be treated with highly efficacious DAAs. (B1)

## HCV laboratory testing before and after SVR

### Serologic assays

Exposure to HCV is determined by testing for anti-HCV antibodies using an approved enzyme (EIA) or chemiluminescent immunoassay (CIA). The presence of anti-HCV antibodies indicates a current or previously resolved HCV infection. The absence of anti-HCV antibodies usually shows that the patient has not been infected. However, anti-HCV antibodies might not be detectable in the first few weeks after the initial infection (window period) in immunosuppressed patients, or in patients who resolve their infection many years later [150, 151].

Several countries in the Asia–Pacific region have developed their own individual testing algorithms for anti-HCV antibody testing. A high signal-to-cutoff ratio for a sample in a specific EIA is highly predictive of an authentic anti-HCV antibody reactive result. The recombinant immunoblot HCV assays recommended for supplemental testing are no longer available. Molecular testing for HCV RNA is currently the only supplemental testing [4, 152].

Recently, rapid assays for detecting HCV antibodies in fingerstick capillary blood and venipuncture whole blood were shown to provide high sensitivity and specificity of >98 % [153]. Given their very high accuracy, convenience, and quick turnaround time, rapid diagnosis tests (RDT) and point-of-care tests (POCT) may be useful in nontraditional settings such as emergency departments, outreach clinics, and community-based organizations.

The currently available HCV core antigen quantitative assay has a sensitivity of 90 % for samples with HCV RNA >10,000 IU/mL and a specificity of near 100 % across HCV GT-1–6 [154]. There is good correlation between HCV core antigen and HCV RNA levels. However, it is probably not suitable for on-treatment monitoring due to its limited sensitivity. The major role these assays might play is in the identification of blood donors in the seroconversion window.

### #9 Consensus statements and recommendations on anti-HCV testing

1. Anti-HCV antibody testing should be performed using approved anti-HCV third- or fourth-generation EIA or CIA. (A1)
2. Samples negative in an approved EIA/CIA can be reported as anti-HCV negative. However, it should be noted that individuals on hemodialysis or coinfected with HIV might be HCV RNA positive but anti-HCV negative. (A1)
3. Samples reactive in an approved single EIA can be reported as anti-HCV positive provided the signal-to-cutoff ratio is sufficiently high to be predictive of a true positive.* (B1)
4. Noninstrumented point-of-care tests (POCT) with rapid anti-HCV testing could serve as first-line screening for hepatitis C (B2).

*The ratio is calculated by dividing the optical density (OD) value of the test sample by the OD of the assay cutoff. An example of the calculation of the ratio: Sample OD = 2.991, cutoff OD = 0.377, therefore S/CO ratio = 7.934. For most standard EIAs (i.e., those not using chemiluminescence), S/CO ratio greater than 3–4 should be indicative of the presence of true anti-HCV antibodies.

### Molecular assays

#### Nucleic acid testing (NAT) for HCV RNA detection

NATs remain the gold standard for diagnosing HCV viremia, determining acute or chronic infection, and monitoring/assessing virologic responses to antiviral therapy. HCV RNA testing should be strongly considered in patients at high risk of infection but who might be anti-HCV negative because they are in the early phase of acute HCV infection or immunosuppression.

The assays used for qualitative HCV RNA testing include endpoint PCR and transcription-mediated amplification (TMA), which have detection limits of 50 and 10 IU/mL, respectively. Although negative TMA results are more predictive of a SVR, a single positive TMA result should be interpreted with caution because patients with positive TMA results may achieve a SVR [155]. Recently, with the introduction of more sensitive quantitative real-time PCR assays, qualitative NATs may be only useful in mass screening of blood donors [156].

HCV viral loads, although not associated with disease activity or progression to chronicity, have been associated with disease progression [157], development of HCC [158], and the treatment outcome of anti-HCV therapy with both interferon-based regimens [159, 160] and new, powerful DAA interferon-free regimens [161]. Monitoring of viral loads during therapy has proven to be useful in individualized HCV therapy. Futility rules and response-guided therapy (RGT) based on in-treatment virologic responses at treatment weeks 4, 8, 12, and 24 could provide information for optimal treatment durations to avoid unnecessary therapy, maximize cost-effectiveness, and minimize adverse events with interferon-based therapy [162–165].

Commercial signal amplification (e.g., branched-DNA assay) and target amplification assays (e.g., real-time PCR assays) are available for quantification of HCV RNA. In-house testing with traditional endpoint PCR for viral loads, which is used widely across the Asia–Pacific region, is not encouraged due to its limited dynamic range compared with commercial, sensitive PCR assays [166]. Such assays should be calibrated to the World Health Organization (WHO) International Standard [167].

With recent advances in RGT strategies and new treatment regimens with DAAs, only assays with a lower limit of quantification of ≤25 IU/mL and a lower limit of detection of 15 IU/mL are recommended. The presence of detectable but not quantifiable HCV RNA is clinically important because this condition reflects true viremia [168]. Therefore, several real-time PCR assays, which have a broad dynamic range of quantification and are sensitive, specific, precise, and reproducible, are preferred [169–172]. In contrast, the bDNA assay, with its low sensitivity, has limited applicability in monitoring treatment.

The traditional primary endpoint of assessing anti-HCV therapy is SVR24 (HCV RNA <50 IU/mL 24 weeks after end of treatment), which indicates that HCV RNA remains negative during long-term follow-up [173]. Recent studies demonstrated that SVR12 (HCV RNA <25 IU/mL 12 weeks after end of treatment) had positive predictive values (PPV) of >98 % for SVR24, regardless of whether therapy was interferon based or DAA interferon free [174, 175]. SVR12 is currently widely used as the primary endpoint of clinical trials and regulatory approval for DAA-containing regimens. SVR12 was suitable for predicting persistent virologic response in almost all cases. In certain DAA-including regimens, SVR12 could not always predict persistent virologic response [176]. Further study of the difference between SVR12 and SVR24 will be needed in interferon-free regimens.

### #10 Consensus statements and recommendations on HCV RNA testing

1. For all samples positive for anti-HCV, HCV RNA should be determined by sensitive nucleic acid testing (NAT). (A1)
2. HCV RNA quantitation should be reported in IU/mL (optionally including copies/mL). DAA-containing regimens should use assays with a lower limit of quantification of ≤25 IU/mL and a lower limit of detection of ≤15 IU/mL. (B1)
3. Monitoring of HCV loads during treatment is important for response-guided therapy to determine futility, treatment protocol, and duration. (A1)

### HCV genotyping

HCV genotyping is helpful in epidemiological studies and necessary for the clinical application of personalized therapy for chronic hepatitis C [177]. Currently, HCV has been classified into seven major GTs, which can be further divided into subtypes [178]. HCV GT-1, GT-2, and GT-3 are widely distributed in the Asia–Pacific region [179], whereas HCV GT-4 and GT-6 are mainly restricted to the Middle East and South East Asia, respectively, and are increasing in prevalence among IDUs [180]. RGT with interferon (IFN)-based therapy is HCV GT specific, and the currently available DAAs are GT and/or subtype specific.

Several methods targeting different regions [5′-untranslated region (UTR), core, NS5A, and NS5B] for HCV genotyping vary from country to country, potentially depending on approval by the relevant health authorities and/or available funding. Methods include direct sequencing, reverse-phase hybridization (e.g., line probe assay), type-specific PCR, restriction fragment length polymorphism analysis after PCR amplification, melting curve analysis after real-time PCR amplification, detection of HCV GT-specific antibodies, and restriction fragment mass polymorphism analysis. Genotyping methods with accurate determination of HCV GT-1 to GT-6 and distinction of HCV GT-1a from GT-1b are required for certain DAA-containing therapies [177].

### #11 Consensus statements and recommendations on HCV genotyping

HCV GT-1a/GT-1b testing is important for assessing treatment regimens and duration and the efficacy of antiviral therapy. (A1)

### Detection of HCV resistance-associated variants (RAVs)

RAVs are naturally occurring HCV quasispecies in the viral life cycle whose frequency mainly depends on their replicational fitness. RAVs may be associated with inferior treatment efficacy, such as the resistance of HCV GT-1a with Q80K mutation to simeprevir, a nonstructural protein 3/4A (NS3/4) protease inhibitor, plus peginterferon/ribavirin [181], and HCV GT-1b with NS5A-L31F/I/M/V and/or NS5A-Y93H mutation resistance to the daclatasvir/asunaprevir all-oral regimen [182]. Excluding patients with preexisting RAVs could enhance treatment efficacy [183].

### #12 Consensus statements and recommendations on HCV RAV detection

Identification of baseline resistance-associated variants is helpful in certain DAA-containing regimens. (B2)

### Host genetic testing

Host single-nucleotide polymorphisms (SNPs) near the interleukin-28B (IL28B, IFN-lambda 3) gene are associated with spontaneous and peginterferon/ribavirin treatment-induced viral clearance in HCV GT-1/GT-4 patients [184, 185] but have a limited role in HCV-2/3 patients [186]. Combined with baseline viral loads, viral kinetics, and previous treatment responses [177], the IL28B genotype could provide important information for decision-making in clinical practice, including: (1) identification of HCV GT-1 treatment-naïve patients who are eligible for 24-week peginterferon/ribavirin (IL28B favorable genotype with baseline viral loads <400,000 IU/mL) [160], (2) early identification of HCV GT-1 nonresponders (IL28B unfavorable genotype with HCV RNA >1000 IU/mL at treatment week 4) [165], and (3) exclusion of retreatment with peginterferon/ribavirin for treatment-experienced HCV GT-1 patients who carry the IL28B unfavorable genotype [187].

### #13 Consensus statements and recommendations on host IL28B genotyping

In interferon-based treatment, IL28B genotyping could help in determining the treatment strategy. (A1)

### Assessment of liver fibrosis

Acute assessment of liver fibrosis is of clinical importance in decision-making, especially in the era of DAA and IFN-free regimens. Patients with cirrhosis with or without other unfavorable factors, such as HCV GT-1a, HCV GT-3, and prior null responses, require intensified treatment [188–191]. Although liver biopsy remains the gold standard to assess liver fibrosis, alternative noninvasive approaches for liver fibrosis have assumed great importance.

Approaches include the following [192–194]:

- Noninvasive imaging (e.g., transient elastography and magnetic resonance elastography/spectroscopy).
- Noninvasive blood marker panels [e.g., aspartate aminotransferase-platelet ratio index (APRI), fibrosis-4 (FIB-4), FibroTest, FIBROSpect II, Hepascore, FibroMeter, and FibroFast]. These tests can provide areas under the curve of >0.7 for identifying clinically significant fibrosis and >0.8 for identifying cirrhosis [195].

Although noninvasive markers and transient elastography are only useful for identifying those patients with no fibrosis or with advanced fibrosis, a stepwise algorithm incorporating noninvasive markers and/or transient elastography may enhance the accuracy of diagnosis and significantly reduce the number of liver biopsies [196, 197]. However, application of the algorithm in clinical practice remains to be validated. Furthermore, accumulating data provide evidence that noninvasive methods for liver fibrosis can be applied at a single point or repeatedly to provide prognostically meaningful distinctions in predicting clinical outcomes, with or without antiviral therapy, in chronic hepatitis C patients [198–200]. A recent study demonstrated that the 3-year evolution of the results of serial noninvasive assessment of hepatic fibrosis strongly predicts the long-term outcome in chronic hepatitis C patients [201].

### #14 Consensus statements and recommendations on noninvasive assessment of fibrosis

Noninvasive methods for assessing liver fibrosis are useful for identifying patients with no/minimal fibrosis or advanced fibrosis and can provide prognostically meaningful distinctions in predicting clinical outcomes in chronic hepatitis C patients before and after successful treatment. A stepwise algorithm incorporating the results of noninvasive methods may enhance the accuracy of diagnosis. Liver biopsies are not always recommended for the diagnosis of hepatitis C or the decision for the treatment of hepatitis C. (B1)

### Laboratory testing after HCV cure

Long-term complications of chronic hepatitis C, including HCC and decompensation, and all-cause mortality are largely reduced after achieving a SVR [202–205]. Nevertheless, SVR patients remain at risk of HCC development, especially those with old age, high baseline gamma-glutamyltransferase levels, advanced liver fibrosis or comorbidity such as diabetes [206–208]. High APRI 6 months after the end of treatment [209] and high posttreatment ALT or AFP levels [210] were independent factors significantly associated with HCC development. These data indicate that regular monitoring for HCC screening and good control of comorbidities after achieving a SVR are mandatory for HCV patients. Older age, male gender, advanced fibrosis, severe steatosis, lower serum albumin levels, non-SVR, and higher post-interferon-treatment ALT or AFP levels are identified as independent factors significantly associated with HCC development [210]. Occurrence of HCC is not a rare event during and after antiviral treatment in older HCV patients [211] or HCV patients with advanced fibrosis [210]. Further studies will be needed regarding this point after interferon-free treatment [212–216].

### #15 Consensus statements and recommendations on laboratory testing after HCV cure

1. Regular HCC screening after achieving a SVR is mandatory for HCV patients at high risk such as patients with advanced fibrosis, older patients, or male patients. (A1)
2. Comorbidities should be evaluated and well controlled to reduce their impact on the disease progression of chronic hepatitis C (B1).

## Electronic supplementary material

Below is the link to the electronic supplementary material.

Supplementary material 1 (DOCX 12 kb)
