# Supplementary material for: APASL consensus statements and recommendations for hepatitis C prevention, epidemiology, and laboratory testing
Source: Hepatol Int. 2016 May 26;10(5):681–701. doi: 10.1007/s12072-016-9736-3 (PMC5003900; doi:10.1007/s12072-016-9736-3)
Supplement: Supplementary file 1 — Supplementary material 1 (DOCX 12 kb) [file 12072_2016_9736_MOESM1_ESM.docx]

**Suppl. Table 1.** Grading of evidence and recommendations (adapted from the GRADE system) [1,2].

| **Grading of evidence** | **Notes** | **Symbol** |
| --- | --- | --- |
| High quality | Further research is very unlikely to change our confidence in the estimate of effect. | A |
| Moderate quality | Further research is likely to have an important impact on our confidence in the estimate of effect and may change the estimate. | B |
| Low or very low quality | Further research is very likely to have an important impact on our confidence in the estimate of effect and is likely to change the estimate. Any estimate of effect is uncertain. | C |
| **Grading of recommendation** | **Notes** | **Symbol** |
| Strong recommendation warranted | Factors influencing the strength of the recommendation included the quality of the evidence, presumed patient important outcomes, and cost. | 1 |
| Weaker recommendation | Variability in preferences and values or more uncertainty make it more likely that a weak recommendation is warranted. Recommendation is made with less certainty; higher cost or resource consumption. | 2 |

Ref

1. Guyatt GH, Oxman AD, Vist GE, Kunz R, Falck-Ytter Y, Alonso-Coello P, et al.GRADE: an emerging consensus on rating quality of evidence and strength of recommendations. BMJ 2008;336:924–926

2. Schunemann HJ, Oxman AD, Brozek J, Glasziou P, Jaeschke R, Vist GE, et al. Grading quality of evidence and strength of recommendations for diagnostic tests and strategies. BMJ 2008;336:1106–1110
